# Study on mechanism of effect of flowing water and transferring heat on rock mass temperature in curved fracture

**DOI:** 10.1038/s41598-023-29992-0

**Published:** 2023-02-20

**Authors:** Junyi Gao, Changyu Lu, Yonggang Zhang

**Affiliations:** 1 Hebei Province Key Laboratory of Sustained Utilization and Development of Water Recourse, Hebei Geo University, Shijiazhuang, 050031 China; 2 School of Architecture and Civil Engineering, Yan’an University, Yan’an, 716000 China; 3 Engineering Research Institute, China Construction Eighth Engineering Division Corp., Ltd., Shanghai, 200122 China

**Keywords:** Environmental sciences, Hydrology, Engineering

## Abstract

Domestically and internationally, the effect of fracture flowing water and transferring heat on the temperature field of surrounding rock in high-level radioactive waste repositories is a popular research area. Compared with straight fracture flowing water and transferring heat, there are few relevant literatures about the heat transfer of curved fracture water flow. Based on the conceptive model of flowing water and transferring heat in curved fractured rock mass, the influence of flowing water and transferring heat in “I”, “L”, , and  shaped fractures on the temperature field of rock mass is calculated by using discrete element program. The findings indicate that: When the model goes into a stable state under four working conditions, the rock on the x = 0–2 m mostly forms a heat transfer path from left to right; the x = 2–4 m primarily forms a heat transfer path from bottom to top, and the temperature gradient reveals that the isotherm of 40–45 °C is highly similar to the shape of four different fractures, indicating that flowing water and transferring heat in the fracture configuration dominate the temperature field of the right side rock mass. The direction of the flowing water and transferring heat of the fracture exerts a dominant effect on the temperature of the rock mass than the length.

## Introduction

**Figure 1:**
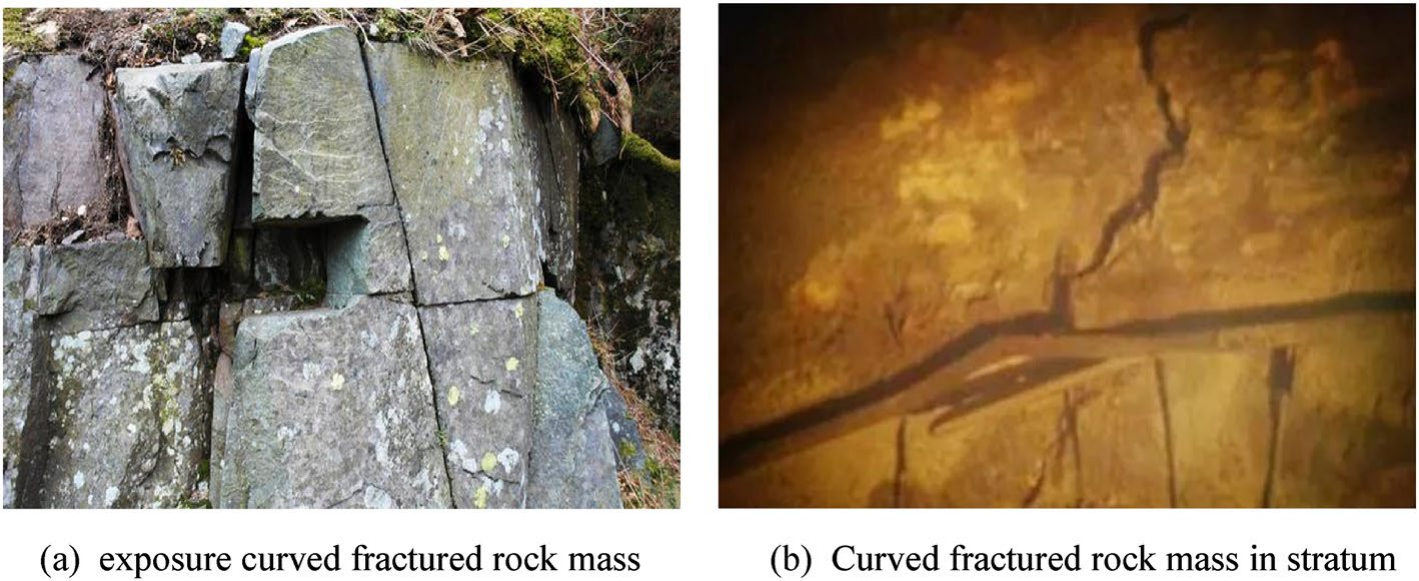
Different rock fracture structures in geological structures.

At present, the coupling of water and heat in fractured rock mass is a hot research topic of domestic and international scholars. There are many areas, such as disposal of high-level radioactive waste, geothermal exploitation engineering, heavy oil exploitation engineering, and so on, all of which are associated with the coupling of water and heat in fractured rock mass and their overall construction. As there are various kinds of fractures in the natural rock mass of the aforementioned projects, new fractures and faults will come about in the initial excavation and drilling process. Different fracture patterns in geological structures are illustrated in Fig. 1. Heat release of sewage tank, hot water injected from geothermal wells and heavy oil wells, and groundwater seepage in fractured rock mass form the temperature effect of seepage and heat transfer. Therefore, the temperature field and water flow field created by the coupling of fracture flowing water and rock transferring heat are directly related to the safe operation throughout the later period of project construction.

Current research on seepage and transferring heat in fractured rock masses focuses mostly on mathematical analysis and numerical simulation. The effect of flowing water and transferring heat in sparse fractures on rock mass temperature was analyzed numerically^1,2^. Conduct the research into the time domain semi-analytical calculation method for seepage and transferring heat in fractured rock mass^3^. Numerical research on the seepage and transferring heat in complex fractured rock mass was conducted^4^. The influence of nonlinear seepage on seepage and heat transport in fractured rock was studied^5^. Experimental and numerical research of flowing water and transferring heat in fissured rock mass was performed^6^. Conduct the study on flowing water and transferring heat model of geothermal reservoir based on discrete fracture network method^7^. Applied to laboratory geothermal systems, a three-dimensional porous elastic simulation analysis of rock mass flow, transferring heat and deformation was carried out^8^. Comprehensively study the influence of thermal disturbance and in-situ stress on heat storage and transferring heat in fissured geothermal reservoirs^9^. Research the single-phase flow and transferring heat in fissured geothermal reservoirs by using a nonlocal continuous approach^10^. The estimation of effective thermal conductivity of rock mass was researched^11^. Research the influence of multi-fissure water flow direction on the near-field temperature of HLW^12^. Study the multi-scale crack roughness and heat transfer of zigzag crack seepage^13,14^. The thermo-hydro-mechanical coupling of fissured rock mass was researched^15–17^. Conduct the distributed thermal research on the characteristics of rock mass^18^. Study the nonlinear heat flow, in-situ stress and heat transfer of the network geometry of fractured geothermal reservoirs^19,20^. Research the stimulation characteristics of hydraulic fracturing reservoirs in enhanced geothermal systems and the stimulation design of enhanced thermal recovery reservoirs in horizontal wells^21–23^. A heat breakthrough of a hydrothermal project in Germany was studied^24^. Research the mechanical properties and perviousness unfolding of salt rock under thermal-hydro-mechanical coupling conditions^25^. In light of this, scholars all over the world have generally studied the hydrothermal coupling of simple straight fractures, while the hydrothermal coupling of complex curved fractures has received few attentions. In practical engineering, due to tectonic stress and human engineering activities, rock cracks are frequently curved. Therefore, it is vital to study the mechanism of the effect of flowing water and transferring heat on rock temperature in curved fracture.

In this paper, the numerical model of the influence of flowing water and transferring heat on the temperature of rock mass in curved fracture is established by using 3DEC program. The influence of flowing water and transferring heat in I-shaped fractures, L-shaped fractures, -shaped fractures and -shaped fractures on rock mass temperature is studied, and the law governing the influence of flowing water and transferring heat in various fracture configurations on rock mass temperature is revealed.

## Discrete element simulation

3DEC is a three-dimensional numerical program based on the discontinuous finite element method. It is a calculation program formed on the basis of two-dimensional UDEC to simulate the response of discontinuous media to static or dynamic loads. The calculation of discontinuities under the condition of setting boundary conditions is mainly inclined to the analysis of rock engineering projects, and various joints and fractures can be easily generated by using Jest command flow. The temperature and the three elements of heat flux are the main manifestations of heat conduction module variables in the 3DEC discrete element program. These variables are relevant to Fourier law of heat conduction and energy balance equation. The differential equation of heat conduction is obtained by combining Fourier law with energy balance equation. The differential equation of thermal transfer can be figured out under specific geometric, boundary, and initial condition. The following dimensionless numbers are used to represent transient thermal transfer.

Natural length:

1 $$Lc = \frac{{V_{s} }}{{A_{s} }}$$

where the characteristic length of solid is expressed by $Lc$ [*m*]; the volume of solid is expressed by $V_{s}$ [*m*^*3*^]; and the surface area of heat exchange is expressed by $A_{s}$ [*m*^*2*^].

Heat diffusion coefficient:

2 $$\kappa = \frac{k}{{\rho C_{v} }}$$

where $\kappa$ is the heat diffusion rate in [m^2^/s]; *k* is the heat conduction in [W/(m·°C)]; *ρ* is the density in [kg/m^3^]; *C*_*v*_ is the specific heat at constant volume in [J/kg·°C].

Natural time:

3 $$t_{c} = \frac{{L_{C}^{2} }}{\kappa }$$

where it indicates the natural time of solid by $t_{c}$ [*s*].

The differential expression of the energy balance is as follows:

4 $$- q_{i,i} + q_{v} = \frac{\partial \zeta }{{\partial t}}$$

where *q*_*i,i*_ is the thermal flux vectors in [W/m^3^]; *q*_*v*_ is the density of volume thermal source in [W/m^3^]; and *ζ* is the amount of thermal stored in a unit volume in [J/m^3^].

Normally, the trade of temperature can also be precipitated via variant of each strength storage and volumetric stress *ε*. And the constitutive heat law related to these arguments may be conveyed as follows:

5 $$\frac{\partial T}{{\partial t}} = M_{th} \left( {\frac{\partial \zeta }{{\partial t}} - \beta_{th} \frac{\partial \varepsilon }{{\partial t}}} \right)$$

where *M*_*th*_ and *β*_*th*_ are material constants; *T* indicates the temperature.

Within this rule, a special example of *β*_*th*_ = 0 and *M*_*th*_ = $\frac{{1}}{{\rho C_{v} }}$ is meditated, in which *ρ* is the solid density of the method in [kg/m^3^], and *C*_*v*_ is the specific heat capacity in [J/kg °C]. It is assumed that the change of strain has little effect on temperature, which is applicable to quasi-static rigid questions including solid and liquid.

6 $$\frac{\partial \zeta }{{\partial t}} = \rho C_{v} \frac{\partial T}{{\partial t}}$$

By replacing Eq. (6) for (4), energy balance formula was yielded.

7 $$- q_{i,i} + q_{v} = \rho C_{v} \frac{\partial T}{{\partial t}}$$

It is worth pointing out that, in principle, all solids and liquids have equal specific heat at constant pressure and constant volume. Therefore, *C*_*v*_ and *C*_*p*_ can be emploied together. According to the principle of the finite difference approximation of the spatial derivative, the number from 1 to 4 embodies the four vertices of the tetrahedron, and the opposite side of the node n is plane n. The merit of superscript (f) is concerned the related variables on plane f.

The temperature in the tetrahedron varies linearly. According to the Gauss divergence theorem, the temperature gradient is represented by the node merit of temperature.

8 $$T,_{j} = - \frac{{1}}{{{3}V}}\mathop \sum \limits_{l = 1}^{4} T^{l} n_{j}^{(l)} S^{(l)}$$

where the exterior unit vector perpendicular to the surface *l* is represented by [*n*]^(*l*)^, the superficial area is represented by *S*, and the tetrahedral volume is represented by *V*.

Node energy balanced equation. The energy-balance Eq. (7) may be represented as:

9 $$q_{i,i} + b* = 0$$

where

10 $$b* = \rho C_{v} \frac{\partial T}{{\partial t}} - q_{v}$$

is the momentary “physical strength” in the formula for mechanical node. Using a tetrahedron analogy, the node thermal $Q_{e}^{n} [w]$ n = 1, 4, in the balance with its thermal flux and physical strength, can be conveyed as:

11 $$Q_{e}^{n} = Q_{t}^{n} - \frac{{q_{v} V}}{{4}} + m^{n} C_{v}^{n} \frac{{{\text{d}}T^{n} }}{dt}$$

where

12 $$Q_{t}^{n} = \frac{{q_{i} n_{i}^{(n)} S^{(n)} }}{3}$$

and

13 $$m^{n} = \frac{\rho V}{{4}}$$

Within this theory, the node form of energy balance equations need exist in each global node, where the sum of equivalent node heats ($- Q_{e}^{n}$) of all tetrahedrons, the applied boundary flux and the node contribution ($Q_{w}^{n}$) of the source is zero.

In the thermal convection module, it is assumed that the solid matrix is impermeable, and the fluid occurs to the rock fractures. As mentioned in the previous section, fluid convection, self- conduction and rock mass conduction are used to make it possible to transfer heat. Generally, the fluid temperature varies from different rock interfaces. According to Newton's cool law, there is a temperature difference between the fracturing fluid and the rock interface, which may cause thermal convection. The rules of thermal convection in rock and fluid are as follows.

Thermal transfers from the plane is represented by the following equations. According to Fourier's law, heat is transmitted through conduction in the fractured fluid.

14 $$q_{f}^{T} = - k_{f}^{T} \Delta T$$

where $q_{f}^{T}$ is the specific heat flows rate of fluid in [W/s^2^], and $k_{f}^{T}$ is heat conductivity of fluid in [W/(m °C)]. The energy balance equation of fluid obeys the equation.

15 $$\rho_{f} c_{f} \frac{{\partial T_{f} }}{\partial t} + \nabla \cdot q_{f}^{T} + \rho_{f} c_{f} q^{f} \cdot \nabla T_{f} + A_{f} h(T_{f} - T_{s} ) = 0$$

where $\rho_{f} c_{f}$ is fluid density [kg/m^3^] multiplied by specific heat [J/(g °C)]; $q^{f}$ is the specific fluid flow rate in [m^2^/s]; $A_{f}$ is a contact area per unit volume of liquid in [m^2^]; h is the thermal conductivity of fluid/rock in [W/(m^2^ °C)]; and *T*_*f*_, *T*_*s*_ are the temperature of liquid and solid block.

For the blocks, the fluid flow was neglected; heat transfer follows Fourier's law. As follows:

16 $$q_{{}}^{T} = - k_{{}}^{T} \Delta T$$

where *q*^*T*^ is specific heat flux rates in [W/s^2^], and *k*^*T*^ is heat conductivity of rock in [W/(m °C)]. The energy balance is

17 $$\rho_{s} c_{s} \frac{{\partial T_{s} }}{\partial t} + \nabla \cdot q_{s}^{T} - A_{s} h(T_{f} - T_{s} ) = 0$$

where $\rho_{{\text{s}}} c_{s}$ is density of solid state [kg/m^3^] multiplied by specific heat [J/(g °C)]; and *A*_*s*_ is contact area of solid per unit volume (from the fluid point of view, both sides are in contact: $A_{s}^{ + }$, $A_{s}^{ - }$, and *A*_*s*_ = $A_{s}^{ + }$ + $A_{s}^{ - }$).

## Computational domain

**Figure 2:**
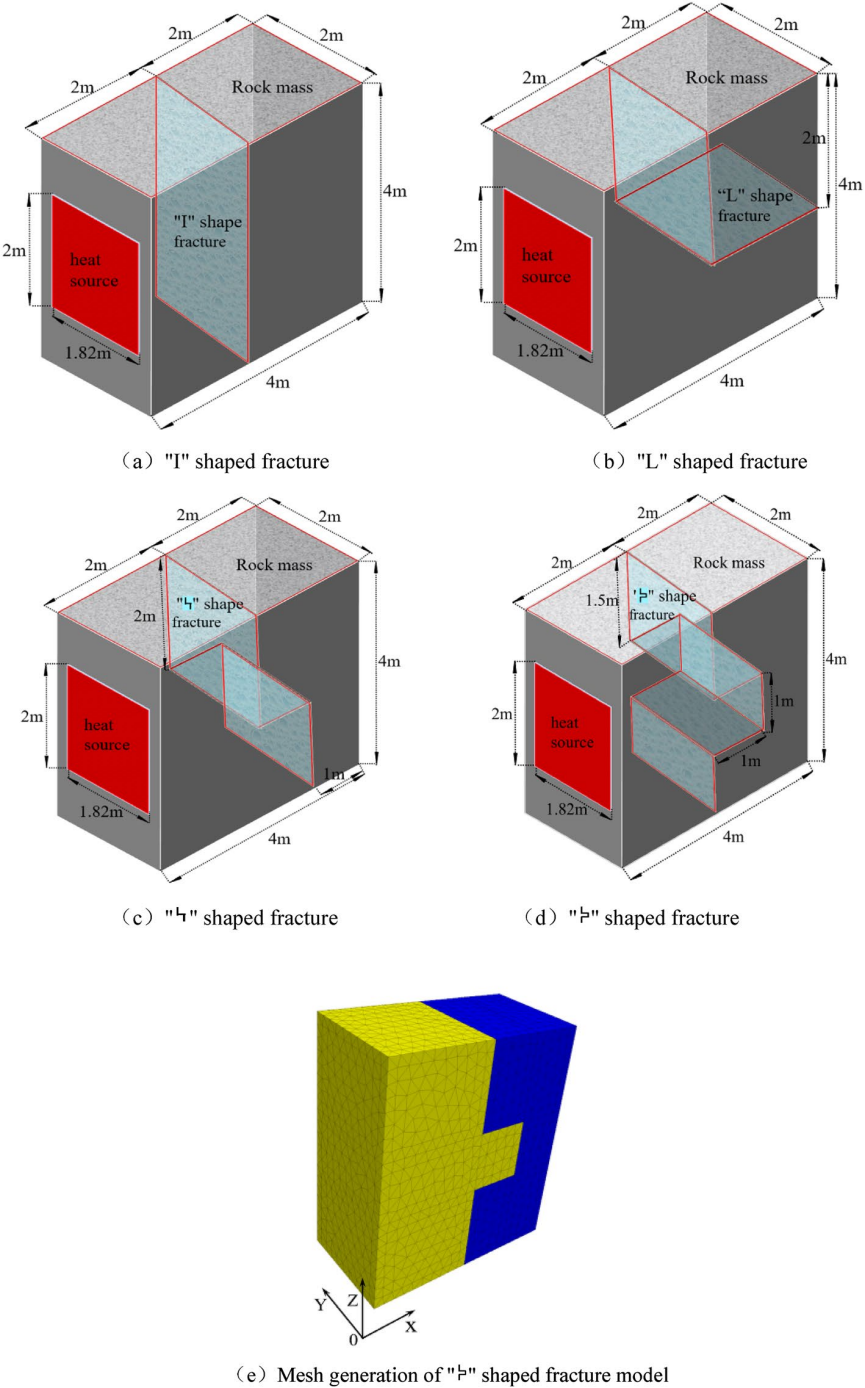
Calculate model dimension and mesh generation (Dimension unit: m).

The calculation model for the effect of flowing water and transferring heat on the rock mass temperature in a curved fracture is 4 m (length) × 2 m (width) × 4 m (height). In the model analysis, it is supposed that the heat source, the simulated waste canister, is positioned in the center of the left side of the model and that its dimensions are 2 m (length) × 1.82 m (width) × 0.01 m (thickness). The distance between the higher and inferior boundaries of the heat source and the higher and inferior boundaries of the model is 1 m, whereas the distance between the left and right boundaries of the heat source to the left and right boundaries of the model is 0.09 m. In this model, the through channels of various fracture types in the near-field rock mass of the reservoir for fracture simulation treatment are arranged in the middle of the left and right sides of the model. Assuming the fracture is smooth and unfilled. The coordinates of temperature observation points A, B, and C in the model are (3.9, 1.0, 3.9), (3.9, 1.0, 2.0), and (3.9, 1.0, 0.1), respectively, that is, the three temperature observation points are located in the upper, middle and lower parts of the right side of the model away from the heat source side. Refer to Fig. 2 for the dimension, location, and grid division of the model. Assuming that the temperature of the heat source is 90 °C, the boundary conditions are as follows: the temperature of the fracture entrance unit is set to normal, the temperature of the fracture exit unit is set to free, and all other surfaces are insulated. At 500 m below the surface, the surrounding rock temperature is around 19 °C, and the model assumes that the initial of rock and fracture water temperature is also 19 °C.

### Parameters and working conditions

The thermophysical parameters of solid and liquid in the numerical model are cataloged in Table 1. Assume that the initial temperature of the surrounding rock and fracture water is 19 °C, and that the convective transferring heat coefficient between rock and water is 30 W/(m^2^ °C).

**Table 1:** Thermo-physical parameters of solid and liquid.

Material	Density/(kg/m^3^)	Specific heat/(J/(g °C))	Coefficient of heat conduction/(W/(m °C))	coefficient of linear thermal expansion/(10^–6^/ °C)
Rock	2700	800	2.3	10
Water	1000	4200	0.6	0.2

Table 2 displays the calculation contents of the model to demonstrate the effect of flowing water and transferring heat in various types of fractures on rock mass temperature. In the model, four types of working conditions are considered. Under the condition that the fracture opening is 1.5 mm, the fracture water flow velocity is 3 mm/s, and the heat release time of the heat source is 242.5 days (the time after the model has reached the steady state), four working conditions of "I" shaped fracture, "L" shaped fracture,  shaped fracture, and  shaped fracture are set respectively to analyze the influence of flowing water and transferring heat in different configurations on the rock mass temperature. The data procured under each condition is made into the temperature field of rock mass and the water temperature time curve at the fracture outlet by post-processing software for comparative analysis.

**Table 2:** Model calculate schemes.

Number	Fracture configuration type	Fracture aperture/mm	Fracture water velocity V_o_/(mm/s)	Heat release time t/d
1	“I”	1.5	3	242.5
2	“L”			
3				
4				

## Results and analysis

### The temperature field of rock mass

**Figure 3:**
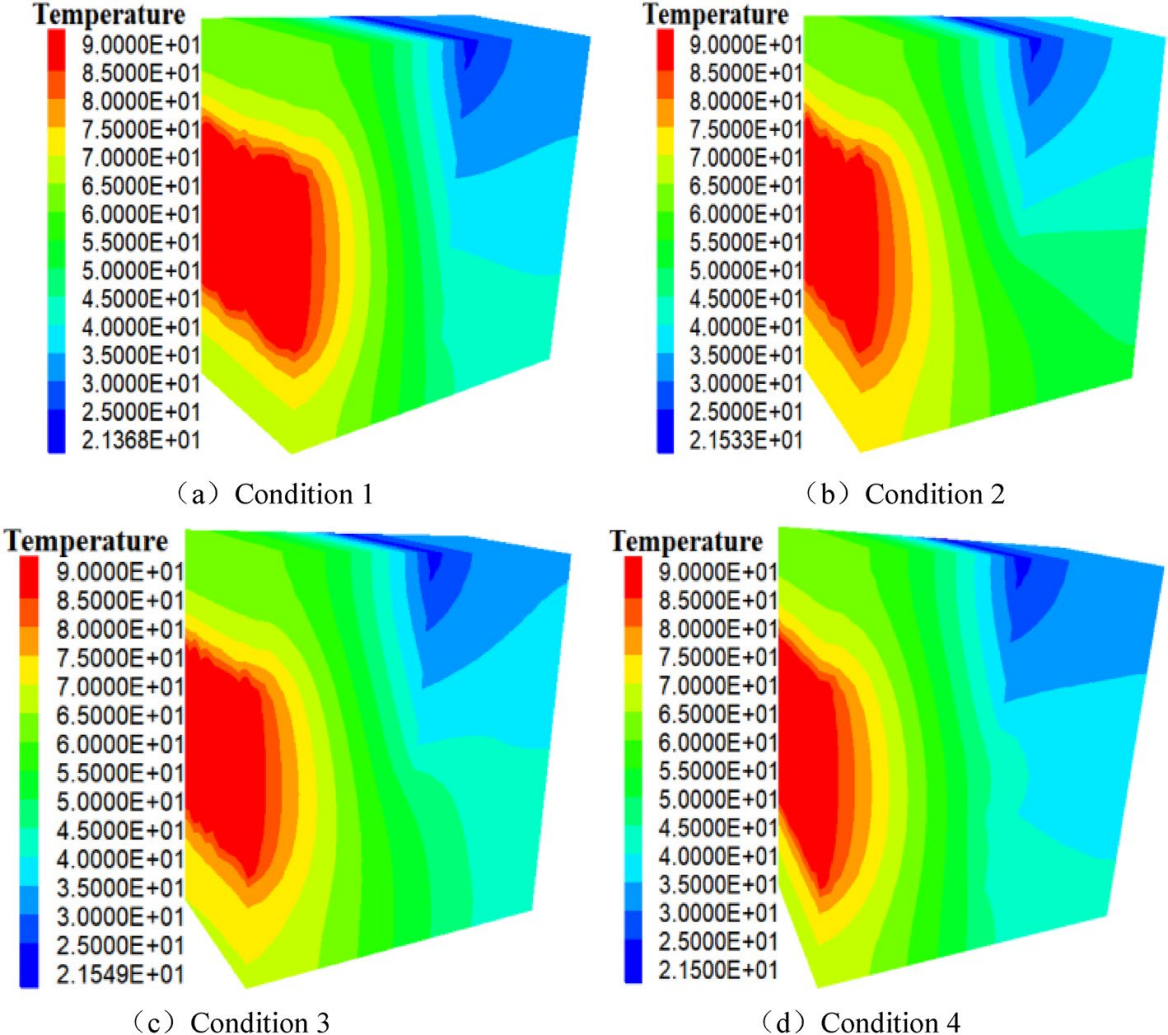
Temperature field of rock mass (unit: °C).

Figure 3 depicts the rock mass temperature field when the model goes into a stable state under the four working conditions.

As shown in Fig. 3a, when the model is a “I” type fracture, the local heat source releases heat to the surrounding area due to the normal temperature water entering the fracture at the upper part of the model, and the heat is transferred to the fracture water flow via rock heat conduction. At this moment, convective heat exchange occurs between the fracture water flow and the rocks on the left side of the model, and some heat is taken away from the fracture water outlet (x = 2 m, z = 0 m). Through the convection heat exchange between the rock and the fracture water flow, heat is transferred to the rock on the right side of the model, and then from the rock to the adiabatic boundary on the right. The “I” shaped fracture primarily functions as a vertical heat barrier to the right side of the model. The gradient of rock temperature between the heat source (with the highest temperature) and the fracture water inlet (with the lowest temperature) is approximately 24.07 °C/m. In the study, the isotherm of 40–45 °C serves as the dividing line (the temperature line in the model changes most obviously). When z = 1.8 m, this temperature line inclines to the lower right corner of the model, forming a local “L” shaped downward warping isotherm. This is because the fracture water absorbs and conducts heat from the upper portion of the model to the lower portion. As shown in Fig. 3b, when the model is an “L” shaped fracture, normal temperature water enters the fracture at the upper portion of the model. After the local heat source releases heat, the heat is transferred to the fractured water flow in the upper part of the model and the rock in the lower part of the fracture through the thermal conduction of the rock. At this time, the fractured water flow conducts heat convectively with the rocks on the left and lower side walls, and part of the heat is taken away from the fractured water outlet (x = 4 m, z = 2 m), and following convection heat exchange, part of the heat is transferred to the rock boundary (upper right side of the model) and the rock boundary on the lower right side of the model. The “L” shaped fracture acts as “L” shaped water flow to prevent heat from transferring the rocks on the right and above the model. The gradient of rock temperature from the site of the rock heat source to the entrance of the fracture is about 24.22 °C/m, and the 40–45 °C isotherm is used as the dividing line to study. When z = 2.0 m, this isotherm which forms a local downward L-shaped isotherm inclines to the lower right corner of the model. As shown in Fig. 3c, when the model is a  shaped fracture, normal temperature fracture water enters the fracture at the upper part of the model. After the local heat source releases heat, the heat is transferred to the fractured water flow of the model through thermal conduction of rock. At this point, the convection heat transfer occurs between the fracture water flow and the rocks on the left side of the model. By convective heat exchange, a portion of the heat is taken away from the fracture water outlet (x = 3 m, z = 0 m) and part of the heat is transferred to the rock boundary on the right side of the model. The  shaped fracture prevents heat from being conducted to the upper side and the right side of the model. From the location of the rock heat source to the entrance of the fracture, the rock temperature gradient is about 24.20 °C/m. The isotherm of 40–45 °C is taken as the dividing line. When z = 2.0 m, this isotherm slightly inclines to the right lower side of the model, forming a  shaped isotherm locally. As may be observed in Fig. 3d, when the model is a  shaped fracture, normal temperature fracture water enters the fracture at the top of the model. After the local heat source releases the heat, it is transferred to the fracture water via heat conduction in the rock. After the fracture water undergoes convection heat exchange with the rock on the left side wall, rock heat conduction transfers the heat to the right boundary of the model. At this time, fracture water and left side wall rock are exchanging heat via convection. A portion of the heat is taken away from the outlet of the fracture water flow (x = 2 m, z = 0 m). A portion of the heat is transferred through convection to the rock on the right side of the model, and subsequently to the adiabatic boundary on the right side of the model. The  shaped fracture blocks the vertical and horizontal heat conduction to the right side and upper part of the model. From the site of the rock heat source to the entrance of the fracture, the temperature gradient is approximately 24.22 °C/m, and the 40–45 °C isotherm is used as the dividing line to study. When z = 1.5 m, this isotherm which forms a local  shaped isotherm inclines to the bottom right corner of the model.

According to the comprehensive comparison in Fig. 3a–d, under the four working conditions (a), (b), (c), and (d), the rock on the left side of the model (x = 0–2 m) predominantly creates a transferring heat path from left to right, and the four heat transfer paths are comparable. The rock on the right side of the model (x = 2–4 m) mostly forms a bottom-up heat transfer path, but its temperature gradient varies under four distinct working conditions. The isotherm of 40–45 °C is taken as the dividing line. Under the four working conditions, the shape of this isotherm closely resembles that of the four fracture configurations of the model, indicating that flowing water and transferring heat in the fracture configuration dominate the temperature field of the rock on the right.

### The temperature field of curved fracture

**Figure 4:**
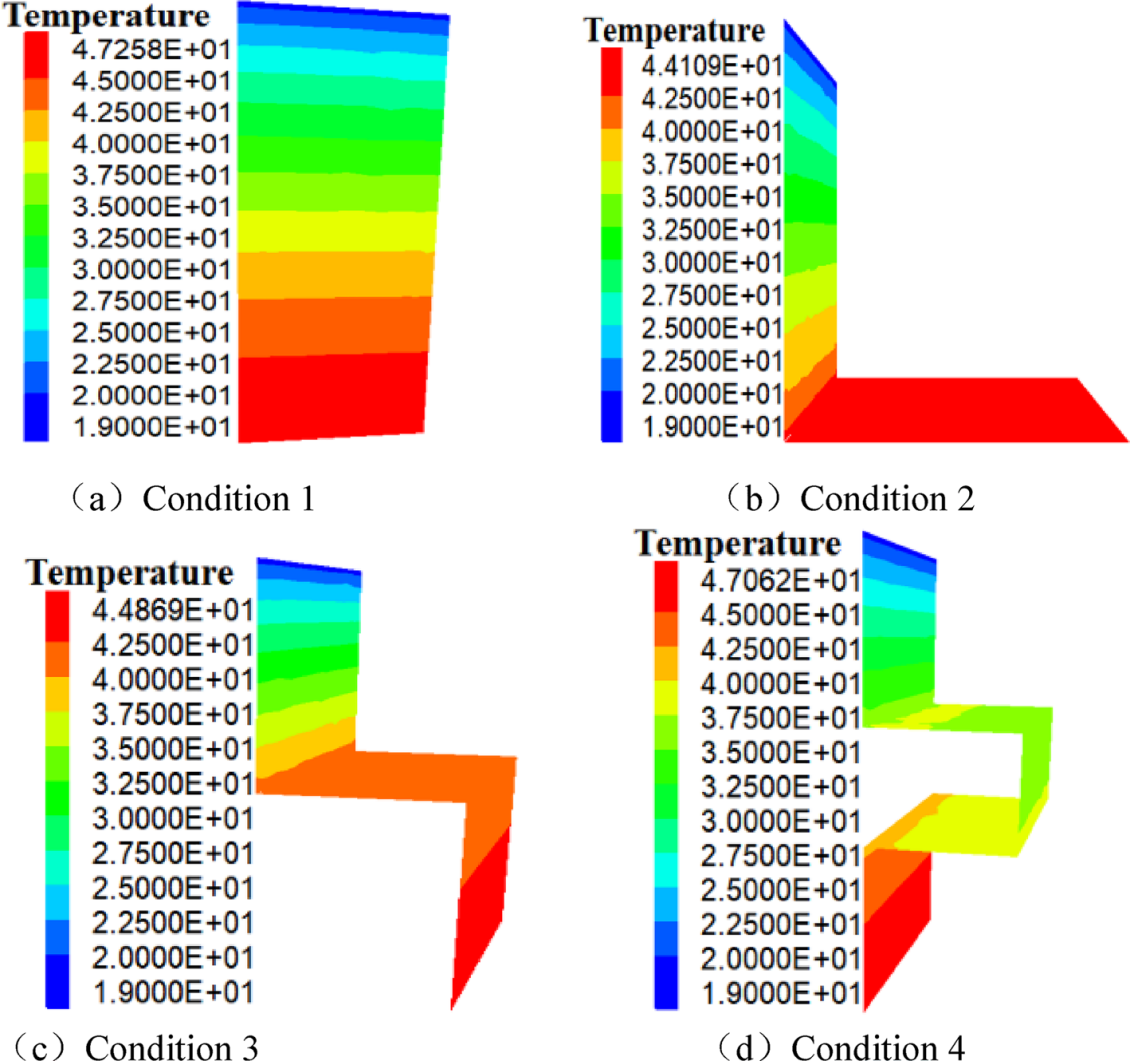
Temperature field of fractured surface water flow (unit: °C).

When the model goes into the stable state, the fracture temperature field is shown in Fig. 4.

As shown in Fig. 4a, when the fracture of the model is “I” shaped, the fracture water flow enters the fracture at the upper part of the model and exits out of the fracture at the lower part of the model. When the model goes into a stable condition, the peak and lowest temperatures of the fracture water flow are approximately 47.26 °C and 19 °C, respectively, and the fracture water flow forms a 7.07 °C/m regular temperature gradient from top to bottom. When the model fracture is “L” shaped, the maximum and minimum temperatures of fracture water are about 44.11 °C and 19 °C, respectively, and fracture water flows from top to bottom and left to right to form a regular temperature gradient, with a temperature gradient of about 6.28 °C/m. As shown in Fig. 4a,b, the heat transfer length of the water in the two fractures is the same, but the heat transfer direction is different, i.e., when the water in the straight fracture becomes a “L” shaped fracture, the temperature gradient of the fracture water decreases by 0.79 °C/m (7.07–6.28 °C/m). As shown in Fig. 4c, when the model fracture is  shaped, the maximum temperature of fracture water is approximately 44.87 °C, and the minimum temperature is around 19 °C. There is a regular temperature gradient from top to bottom, left to right, and top to bottom, which is approximately 5.17 °C/m. According to the comparison of Fig. 4b,c the heat transfer path of the fractured water flow in condition 2 is 1 m shorter than that in condition 3, and the heat transfer direction and outlet location are different. Thus, the fracture water temperature gradient decreases by 1.11 °C/m (6.28–5.17 °C/m) when the “L” shaped fracture is changed into an “I” shaped fracture. As shown in Fig. 4d, when the model fracture is  shaped, the maximum temperature of fracture water is around 47.06 °C and the minimum temperature is approximately 19 °C. The fracture water flow forms a regular temperature gradient from top to bottom, from left (right) to the right (left), which is about 4.68 °C/m. According to (c) and (d), the heat transfer path of fracture water flow in working condition 3 is 1 m shorter than that in working condition 4, and the heat transfer direction and outlet location are different. In other words, when the fracture changes from  shaped fracture to  shaped fracture, the temperature gradient of fracture decreases by 0.49 °C/m (5.17–4.68 °C/m).

Under the four working conditions depicted in Fig. 4a–d, the temperature gradient of the water flow on the cross section is 7.07  °C/m, 6.28 °C/m, 5.17  °C/m, and 4.68 °C/m, respectively. In general, water flows from top to bottom, left to right, and right to left, on the fracture surface, providing a relatively regular temperature gradient. When the “I” shaped fracture transforms into the “L” shaped fracture (the length does not change), the “L” shaped fracture transforms into the  shaped fracture (the length is reduced by 1 m), and the  shaped fracture transforms into the  shaped fracture (the length is reduced by 1 m), the heat flux density and the heat conduction rate of the crack water drop.

### The temperature of rock observation point

**Figure 5:**
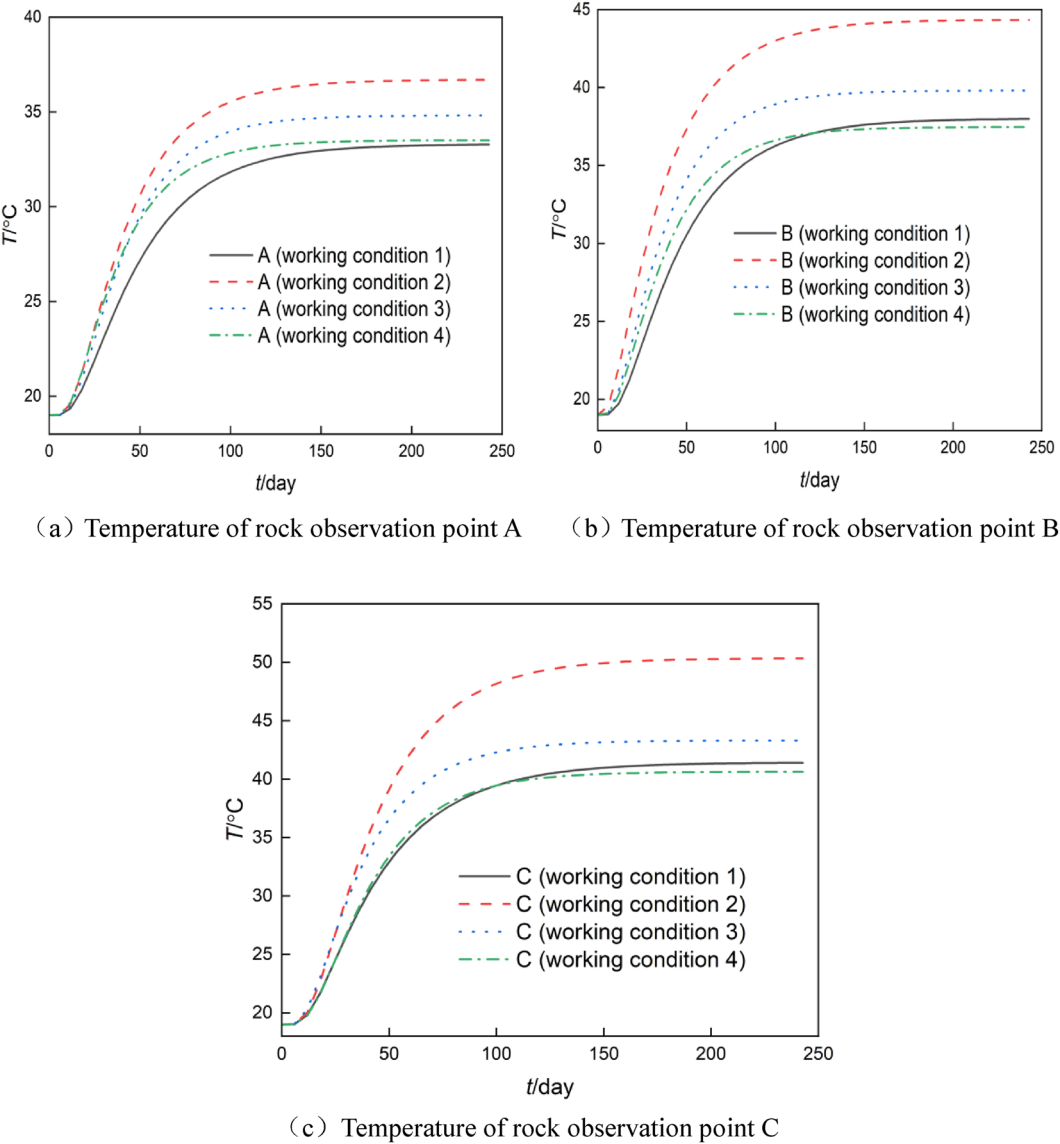
Temperature–time curve of rock observation points A, B, and C.

The temperature of rock observation point under four working conditions is shown in Fig. 5.

It can be seen from Fig. 5a that under four working conditions, the highest temperature at the observation point of “L” shaped fracture (working condition 2) is 36.69 °C, the temperature at the observation point of  shaped fracture (working condition 3) is 34.81 °C, the temperature at the observation point of  shaped fracture (working condition 4) is 33.50 °C, and the lowest temperature of “I” shaped fracture (working condition 1) is 33.27 °C. The maximum temperature at observation point A is the result of the combined action of flowing water and transferring heat from the vertical fracture portion of the “L” shaped fracture to the right rock and from the horizontal fracture portion to the rock above the fracture. The lowest temperature at observation point A is attributed to the vertical fracture water flow in the “I” shaped fracture carrying the majority of heat to the underside of the model. As depicted in Fig. 5b, under the four working conditions, the highest temperature of the “L” shaped fracture observation point (working condition 2) is 44.33 °C, the temperature of the  shaped fracture observation point (working condition 3) is 39.81 °C, the temperature of the “I” shaped fracture observation point (working condition 1) is 37.98 °C, and the lowest temperature of the  shaped fracture observation point (working condition 4). The temperature of observation point B is the highest, because it is located near the outlet of the “L” shaped fracture. The heat carried in water has a greater influence on the temperature at observation point B than that of rock. The lowest temperature of observation point B is due to the vertical water flow in the middle of the  shaped fracture getting distant from the heat source. As can be seen from Fig. 5c, under four working conditions, the highest temperature of the “L” shaped fracture observation point (working condition 2) is 50.33 °C, the highest temperature of the  shaped fracture observation point (working condition 3) is 43.3 °C, the lowest temperature of the “I” shaped fracture observation point (working condition 1) is 41.4 °C, and the lowest temperature of the  shaped fracture observation point (working condition 4). The highest temperature at observation point C is caused by the heat transfer from the heat source to the rock on the right side of the model and the heat transfer from the horizontal portion of the water flow to the lower rock in the “L” shaped fracture. The lowest temperature at observation point C is caused by the water flow in the vertical portion of the middle of the  shaped fracture getting close to the heat source.

The temperatures of the three observation points of “L” shaped fracture (working condition 2) are the highest among the four working conditions (a), (b), and (c), followed by those of the three observation points of  shaped fracture (working condition 3). Due to their similar fracture structures, the temperature at the observation point of "I" shaped fracture (working condition 1) is identical to that of  shaped fracture (working condition 4), except that  shaped fracture has 2 m longer in horizontal heat transfer paths than “I” shaped fracture. Due to the combined effects of heat transfer of fracture water and heat conduction of rock, the temperature is higher at the downstream observation site than that at the upstream observation point.

### The water temperature at fracture exit

**Figure 6:**
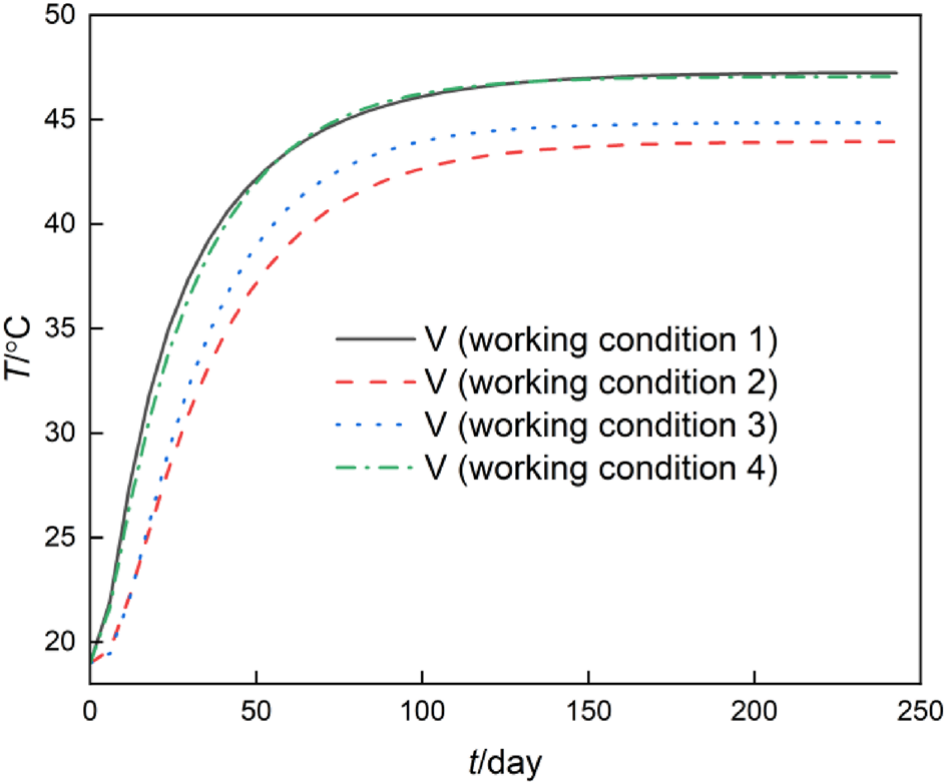
Water temperature–time curve at the fracture exit.

Under the four working conditions, the water temperature–time curve at the fracture exit is shown in Fig. 6.

As shown in Fig. 6 that under the four working conditions, the highest water temperature at the exit of the "I" shaped fracture (condition 1) is approximately 47.24 °C, while the second highest water temperature at the exit of the  shaped fracture (condition 4) is approximately 47.06 °C, and the water-time curves of the two are essentially coincident from the beginning to the end. This is because even though the heat transfer distance between the two horizontal fractures in the  shaped fracture is 2 m longer than that of the “I” shaped fracture, the vertical non-overlapping fracture is only 1 m, and the water flow in the horizontal fracture transfers heat to the lower right and lower left sides of the model. So it has little impact on the water temperature at the fracture exit. The medium–high water temperature at the exit of the  shaped fracture (condition 3) is around 44.86 °C, whereas the lowest water temperature at the exit of the “L” shaped fracture (condition 2) is approximately 43.95 °C. This is because the “L” shaped fracture bends 90° to the right side of the model at (x = 2 m, z = 2 m), and the exit gets increasingly distant from the heat source, resulting in the lower and lower outlet temperature. The outlet water temperature of "I" shaped fracture (condition 1) and  shaped fracture (condition 4) is approximately 3.11 °C (47.06–43.95 °C) higher than that of "L" shaped fracture (condition 2), and the water temperature at the exit decreases by around 6.61%. Under these four conditions, the model goes into a stable condition in approximately 150 days.

According to the overall comparison of Figs. 3, 4, 5, and 6, the influence of fracture flowing water and transferring heat direction on rock mass temperature predominates over the influence of length of fracture flowing water and transferring heat on rock mass temperature.

## Conclusions

In this paper, the 3DEC discrete element program is utilized to analyze the hydrodynamic and thermo-dynamic coupling in four distinct types of fractured rock mass with diverse structures. Under the same other conditions, the effect of flowing water and transferring heat in four types of fractured rock mass with distinct structures on the temperature field of rock mass was analyzed. The results indicate:

1. Under these four conditions, the rock on the x = 0–2 m predominantly creates a transferring heat path from left to right. The rock on the x = 2–4 m primarily forms a heat transfer path from bottom to top, and the four working conditions produce distinct temperature gradients. The 40–45 °C isotherm is used as the dividing line between the research objects, and the four working conditions demonstrate that the shape of the isotherm is highly similar to the four different configurations of the model, indicating that flowing water and transferring heat in the fracture configuration dominates the temperature field of the right rock mass.
2. Under these four conditions, the temperature gradients of the water flow of the fracture are 7.07 °C/m, 6.28 °C °C/m, 5.17 °C/m, and 4.68 °C/m, respectively. Generally, the water flow on the fracture surface generates the regular temperature gradients from top to bottom and from left to right. The heat flux density and the thermal conductivity of fracture water gradually diminish.
3. In the four working conditions, the temperature of the three observation points of the “L” shaped fracture is the highest, followed by the temperature of the three observation points of the  shaped fracture. Due to their similar fracture structures, the temperature at the observation point of “I” shaped fracture is identical to that of  shaped fracture, except that  shaped fracture has 2 more meters than “I” shaped fracture in horizontal heat transfer paths. Due to the combined action of heat transfer of fracture flowing water and transferring heat of rock, the temperature of fracture water flow is higher at the downstream observation site than that of the upstream observation point. In comparison to the effect of heat transfer length and direction of fractured water flow on rock mass temperature, the effect of heat transfer direction of fractured water flow is dominant.

## Data Availability

The datasets generated and/or analyzed during the current study are not publicly available but are available from the corresponding author on a reasonable request.

## References

[1] Gao J, Lei H, Yang H (2020). Numerical analysis of water flow and heat transfer influenceon temperature in plutones with sparse orthogonal and non-orthogonal fracture. Uranium Geol..

[2] Gao J, Xiang Y (2017). Numerical analysis on crossed water flow and heat transfer on the temperature of fractured rocks. Chin. J. Underground Space Eng..

[3] Liu D, Xiang Y (2020). Temporal semi-analytical method for water flow and heat transfer in fractured rocks. J. Central South Univ..

[4] Shao Y (2021). Numerical study on coupling of seepage and heat transfer in 3d complex fractured rock masses. Chin. J. Underground Space Eng..

[5] Yao C (2020). Effect of nonlinear seepage on flow and heat transfer process of fractured rocks. Chin. J. Geotech. Eng..

[6] Tavakkoli OY, Akin S (2021). Experimental and numerical study of flow and thermal transport in fractured rock. Heat Mass Transfer..

[7] Lee T (2018). Development of fluid flow and heat transfer model in naturally fractured geothermal reservoir with discrete fracture network method. Geosci. J..

[8] Gao Q, Ghassemi A (2020). Three-dimensional thermo-poroelastic modeling and analysis of flow, heat transport and deformation in fractured rock with applications to a lab-scale geothermal system. Rock Mech. Rock Eng..

[9] Sun Z (2021). Combined effects of thermal perturbation and in-situ stress on heat transfer in fractured geothermal reservoirs. Rock Mech. Rock Eng..

[10] Vasilyeva M (2019). Upscaling of the single-phase flow and heat transport in fractured geothermal reservoirs using nonlocal multicontinuum method. Comput. Geosci..

[11] Li Z (2021). Effective thermal conductivity estimation of fractured rock masses. Rock Mech. Rock Eng..

[12] Gao J (2021). Analysis of the influence of the multi-fracture water flow and heat transfer direction on the near-field temperature of high-level radioactive waste repository. Arab. J. Geosci..

[13] Tan J (2021). Multiscale roughness influence on hydrodynamic heat transfer in a single fracture. Comput. Geotech..

[14] Ma Y (2022). Experimental study of heat transfer between fluid flowing through fracture surface with tortuous seepage path. Renew. Energy.

[15] Zhou X, Du E, Wang Y (2022). Thermo-hydro-chemo-mechanical coupling peridynamic model of fractured rock mass and its application in geothermal extraction. Comput. Geotech..

[16] Pandey S, Chaudhuri A, Kelkar SA (2017). Coupled thermo-hydro-mechanical modeling of fracture aperture alteration and reservoir deformation during heat extraction from a geothermal reservoir. Geothermics.

[17] Jiao H (2022). Investigation of thermal-hydro-mechanical coupled fracture propagation considering rock damage. Comput. Geosci..

[18] Klepikova M, Brixel B, Roubinet D (2022). Analysis of thermal dilution experiments with distributed temperature sensing for fractured rock characterization. J. Hydrol..

[19] Xiong F (2022). Heat extraction analysis for nonlinear heat flow in fractured geothermal reservoirs. Comput. Geotech..

[20] Sun Z (2020). Joint influence of in-situ stress and fracture network geometry on heat transfer in fractured geothermal reservoirs. Int. J. Heat Mass Transf..

[21] Cheng Y (2021). Investigation on reservoir stimulation characteristics in hot dry rock geothermal formations of china during hydraulic fracturing. Rock Mech. Rock Eng..

[22] Krzaczek M (2020). Simulations of hydro-fracking in rock mass at meso-scale using fully coupled DEM/CFD approach. Acta Geotech..

[23] Zhang Y (2022). Reservoir stimulation design and evaluation of heat exploitation of a two-horizontal-well enhanced geothermal system(EGS) in the Zhacang geothermal field, Northwest China. Renew. Energy.

[24] Fadel M (2022). Causes of a premature thermal breakthrough of a hydrothermal project in Germany. Geothermics.

[25] Lyu C (2022). Mechanical characteristics and permeability evolution of salt rock under thermal-hydro-mechanical (THM) coupling condition. Eng. Geol..

